# Intratumoral Fc-optimized agonistic CD40 antibody induces tumor rejection and systemic antitumor immunity in patients with metastatic cancer

**DOI:** 10.21203/rs.3.rs-4244833/v1

**Published:** 2024-06-03

**Authors:** Juan C. Osorio, David A. Knorr, Polina Weitzenfeld, Ning Yao, Maria Baez, Meghan DiLillo, Jahan Rahman, Jacqueline Bromberg, Michael A. Postow, Charlotte Ariyan, Mark E. Robson, Jeffrey V. Ravetch

**Affiliations:** 1 Laboratory of Molecular Genetics and Immunology, Rockefeller University, New York, NY; 2 Department of Medicine, Memorial Sloan Kettering Cancer Center, New York, NY; 3 Department of Medicine, Weill Cornell Medicine, New York, NY; 4 Department of Epidemiology and Biostatistics, Memorial Sloan Kettering Cancer Center, New York, NY; 5 Department of Surgery, Memorial Sloan Kettering Cancer Center, New York, NY; 6 Department of Surgery, Weill Cornell Medicine, New York, NY; 7 Current address: Regeneron, Inc., Tarrytown, NY, USA

## Abstract

While CD40 agonism is an attractive approach for activating antigen-presenting cells and initiating antitumor responses, previous attempts have encountered limited clinical efficacy coupled with toxicity. We previously demonstrated that interactions between the antibody Fc domain and the inhibitory receptor FcγRIIB are critical for enhanced antitumor activity. Here, we present the results of a phase 1 study on intratumoral administration of an anti-CD40 agonistic antibody (2141-V11) Fc-engineered to enhance FcγRIIB binding. Primary endpoints included safety, maximum tolerated dose (MTD), and recommended phase 2 dose. Secondary objectives included preliminary clinical activity and correlative studies from biospecimens. 2141-V11 was well-tolerated without dose-limiting toxicities and MTD was not reached. In ten evaluable patients with metastatic cancer, the overall response rate was 20%, with complete responses in two patients (melanoma and breast carcinoma) and stable disease in six patients. 2141-V11 induced tumor regression in injected and non-injected lesions, with increased leukocyte infiltration and tertiary lymphoid structures (TLS) formation in post-treatment biopsies. In a humanized mouse model for CD40 and FcγRs, 2141-V11 induced TLS formation in mice bearing orthotopic breast carcinoma, correlating with local and abscopal antitumor effects, systemic immune activation, and immune memory. These findings support the safety and efficacy of 2141-V11, warranting phase 2 studies and suggesting a unique mechanism of action for this Fc-enhanced immunotherapy (NCT04059588).

Blocking inhibitory immune receptors with anti-PD-1/PD-L1 and anti-CTLA-4 antibodies (Abs) is now established as an effective therapeutic strategy for several malignancies^1–3^. However, the benefit of these therapies remains limited to a small subset of patients. Activating the immune system through stimulatory pathways represents a promising alternative and complementary approach. In particular, the stimulatory receptor CD40 plays a central role in promoting antitumor immunity and developing tumor-specific T-cell responses^4^.

CD40 is a member of the tumor necrosis receptor superfamily, expressed predominantly on antigen-presenting cells (APCs) such as dendritic cells (DCs), B-cells and macrophages. When CD40 binds to its ligand, CD40 ligand (CD154), it trimerizes to initiate downstream signaling to activate APCs^5^ (Scheme 1a). This activation is critical for promoting antigen presentation by DCs, which is crucial step to induce effective T-cell responses^6^. Engagement of CD40 can be achieved not only through its cognate ligand but also through agonistic Abs, which promote APC maturation and activation, resulting in T-cell responses and elimination of tumor cells^7^.

Several first-generation CD40 agonist Abs were developed and tested in patients with cancer. However, they demonstrated dose-limiting systemic toxicities (mainly thrombocytopenia and transaminitis) and had limited clinical benefit^8–14^. We have previously demonstrated that the in vivo activity of agonistic anti-CD40 Abs relies on interactions between the Ab fragment crystallizable (Fc) domain and the inhibitory Fc-gamma receptor FcγIIB, resulting in optimal trimerization and agonistic signaling^15–18^ (Scheme 1b). Based on these findings, we engineered 2141-V11, a human IgG anti-CD40 agonistic Ab, which had previously been evaluated in several phase 1 studies as an IgG2 antibody (clone CP-870,893)^19,20^, with point mutations in its Fc portion to selectively increase binding affinity to FcγRIIB^15^. Using an immunocompetent mouse model humanized for both CD40 and all human FcγRs (hCD40/hFcγR mice), we demonstrated that 2141-V11 has enhanced immune stimulatory activity in vivo, resulting in enhanced DC activation and CD8 anti-tumor immunity when compared to other clinical CD40 Abs lacking selective FcγRIIB binding across several tumor models^15^. Studies in our hCD40/hFcγR mouse model also demonstrated that systemic administration of 2141-V11 was associated with increased levels of toxicity (thrombocytopenia and transaminitis)^18^. Importantly, these toxicities were circumvented by intratumoral (IT) administration, allowing for effective *in situ* vaccination effect with systemic antitumor activity and minimal toxicity.

These studies provided the mechanistic foundation for our first-in-human investigator-initiated study (NCT04059588), assessing the safety and tolerability of IT delivery of 2141-V11 in patients with solid tumors locally advanced or metastatic to the skin amenable to IT injection. By optimizing the Fc portion of the CD40 agonistic Ab, and by switching the administration route from systemic to IT, we have addressed the challenges that have led to poor performance of prior anti-CD40 agonistic Abs. The primary endpoint was to determine the maximum tolerated dose (MTD) and the recommended phase 2 dose (RP2D) of 2141-V11. Secondary objectives include evaluating pharmacokinetics and preliminary clinical activity. Immune profiling of biospecimens obtained from patients that achieved complete responses (CR) and in vivo mechanistic experiments using our hCD40/hFcγR mice complement these studies, providing further characterization of the mechanism of action of 2141-V11.

## Results

### Preclinical toxicology and determination of human dosing of 2141-V11

**Extended data Fig. 1:**
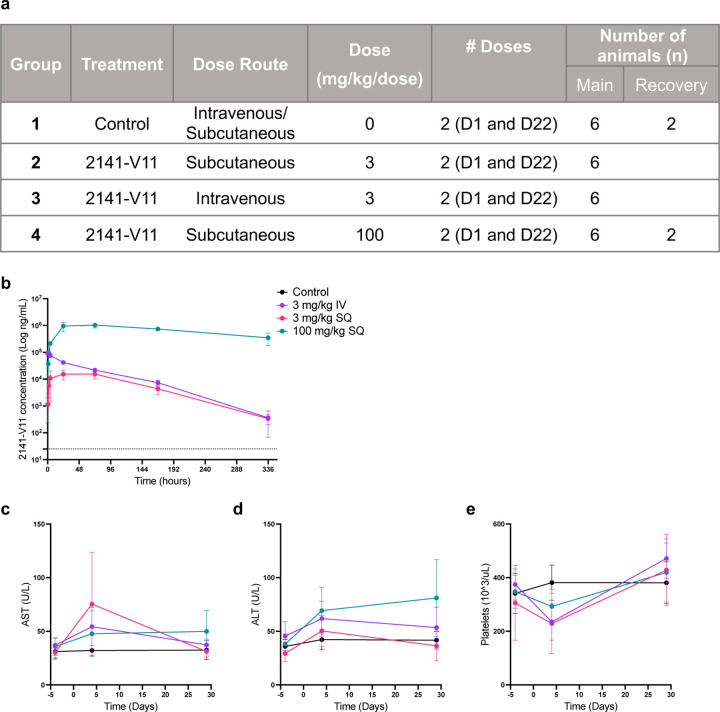
Preclinical pharmacokinetics and toxicology in non-human primates. **a,** Experimental design of studies in non-human primates (NHP) to determine pharmacokinetics and toxicity of 2141-V11 when given by subcutaneous (SQ) or intravenous (IV) injection twice (days 1 and Day 22) to cynomolgus monkeys, and to evaluate the potential of reversibility of any findings. **b,** PK profiles at different doses following a single IV or SQ administration of 2141-V11 (n=24) in cynomolgus monkeys. Dashed line indicates lower limit of quantification (25 ng/mL). Statistical average value and standard deviation are shown. **c,** Levels of AST **d,** ALT **e,** and platelets following a single IV or SQ administration of 2141-V11 (n=24). Statistical average value and standard deviation are shown.

Several pre-clinical assessments were performed to identify a safe initial dose for human trials, aiming to minimize toxicity risk and limit the exposure of subjects to ineffective doses. Pharmacokinetics and toxicology studies were performed in non-human primates (NHP) receiving subcutaneous (SC) or intravenous (IV) 2141-V11 (Extended data Fig. 1). 2141-V11 was detected in serum samples from NHP, with concentration versus time profiles consistent with the route of administration, and peak serum concentrations between 24 and 72 hours (Extended data Fig. 1b). No major adverse events were observed in NHP, with no unscheduled deaths, changes in body weight, adverse clinical signs, or local reactions to SC or IV administration of 2141-V11 during the study period. Further, no major changes in liver enzyme levels or platelets were observed (Extended data Fig. 1c-1e). Based on these results, the MTD of 2141-V11 in NHP was 3 mg/kg/dose and 100 mg/kg/dose when administered IV and SC, respectively.

In contrast to the lack of toxicity observed in NHP, we previously evaluated the pharmacokinetics and toxicity of 2141-V11 in a mouse model that recapitulates the human expression of CD40 and FcγRs instead of the murine versions (hCD40/hFcγR mice)^18^. Increasing doses of systemic 2141-V11 led to worsening thrombocytopenia and transaminitis in hCD40/hFcγR mice^18^, consistent with reports from previous clinical studies^8,21^. In this model, we established that the MTD of systemic and IT administration of 2141-V11 were 0.1 mg/kg and 0.4 mg/kg, respectively^18^. These large differences in recommended doses and toxicity between NPH and hCD40/hFcγR mice can be attributed to the weak binding of NHP FcγRs to the Fc that we specifically engineered to enhance binding to human FcγRIIB. We found this enhancement to be an essential component for the potency and toxicity of 2141-V11^15^, further illustrating the importance of matching antibody Fc domains with cognate, species-matched Fc receptors. Consequently, NHP data was not used for calculating the proposed human dose; instead, the hFcγR/hCD40 mouse model provided the best predictive preclinical model. We determined that the initial, human-equivalent IT dose was 0.03 mg/Kg. A 3-fold safety factor was then applied, resulting in 0.01 mg/kg. Thus, a 70 kg individual would receive a fixed IT starting dose of 0.7 mg, which was subsequently dose-escalated in a 3 + 3 design to doses of 2.0 mg, 7.0 mg, and 10.0 mg.

### Study design and patient characteristics:

**Figure 1.**
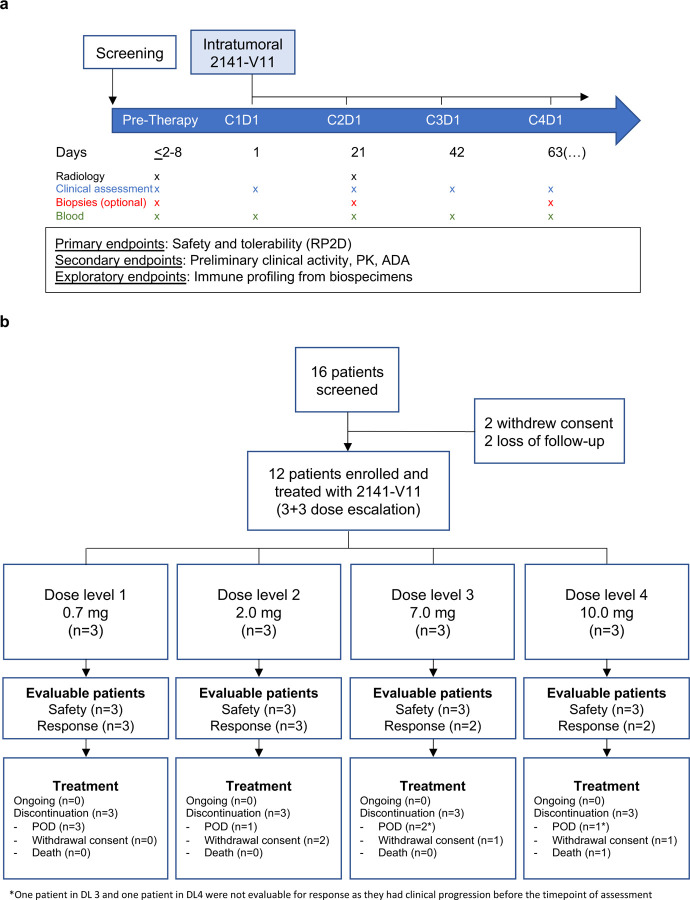
Clinical trial design and patient flow. **a,** Patients with solid tumors locally advanced or metastatic to the skin were screened to receive a fixed dose of intratumoral 2141-V11 every three weeks, on day 1 of a 3-week cycle. Dose levels started at 0.7 mg and escalated in a 3+3 design. Primary, secondary, and exploratory objectives are indicated. **b,** Sixteen patients underwent full screening and 12 received at least one dose of 2141-V11. Four patients dropped out before receiving 2141-V11 due to withdrawal of consent (n=2) or loss of follow-up (n=2). Twelve were treated with at least one dose of 2141-V11 and allocated in four dose levels (0.7; 2.0; 7.0; and 10 mg). C: Cycle. D: Day. DL: Dose level.

This phase 1 study (NCT04059588) enrolled patients with metastatic solid tumors and identifiable metastatic lesions in the skin, subcutaneous tissue, or lymph node amenable to IT injection (Fig. 1a). The study was conducted from January 2020 to January 2024. The first patient was enrolled on 16 January 2020 and the last on 28 July 2022. Baseline patient characteristics are presented in Table 1. A total of 12 patients were enrolled in the study and received treatment across four dose levels (DL1=0.7 mg; DL2=2.0 mg; DL3=7.0 mg; DL4=10.0 mg) (Fig. 1b). Among them, seven (58.3%) had breast cancer, three (25%) had melanoma, and two (16.7%) had renal cell carcinoma. The median age was 64 years (range 42–89), the median time from diagnosis was five years (range 1–29), and the median number of previous lines of therapy was five (range 0–14). Prior lines included immunotherapy in seven (58.3%) patients, and systemic chemotherapy in eight (66.6%) patients (Extended data Table 1).

### Safety, pharmacokinetics, and immunogenicity of 2141-V11

**Figure 2.**
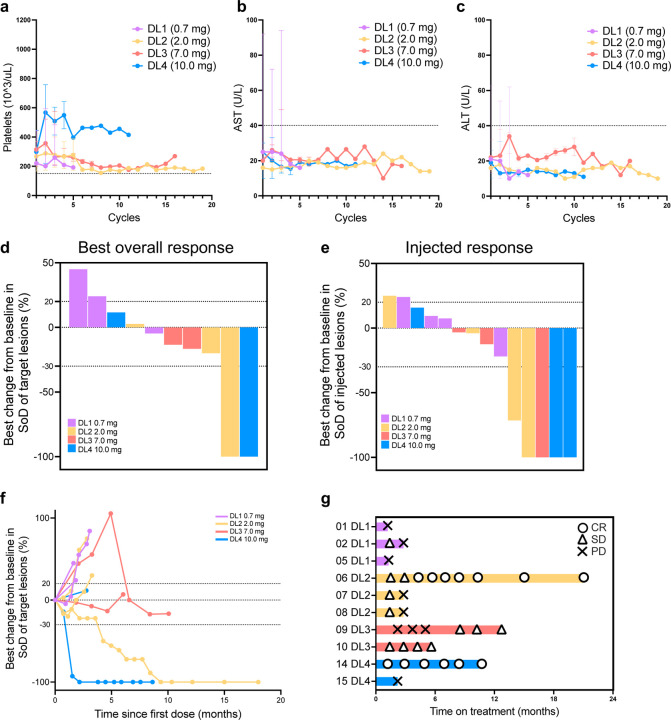
Safety and preliminary clinical activity of 2141-V11. **a,** Platelet count (10^3^/uL) before and after administration of intratumoral 2141-V11. Median value and range from patients treated within the same dose level at each cycle of treatment are shown. Dashed line indicates lower limit of normal level (150 ×10^3^/uL). **b-c,** AST, and ALT levels (U/L) before and after administration of intratumoral 2141-V11 at the same timepoints described above. Dashed lines indicate upper limit of normal levels (40 U/L). **c,** Waterfall plot displaying best percent change from baseline in sum of lesion diameters (SoD) of target lesions among patients that were at different dose levels (0.7 mg violate bars; 2.0 mg yellow bars; 7.0 mg salmon bars; 10 mg blue bars) and were evaluable for antitumor activity (n=10). **d,** Waterfall plot displaying best percent change from baseline in sum of lesion diameters (SoD) of injected target lesions across different dose levels (0.7 mg violate bars; 2.0 mg yellow bars; 7.0 mg salmon bars; 10 mg blue bars). **e,** Spider plot displaying percent change from baseline in sum of diameters (SoD) of target lesions over time in the study. Best response of patients at different dose levels (0.7 mg violate lines; 2.0 mg yellow lines; 7.0 mg salmon lines; 10 mg blue lines) are indicated. **f,** Swimmer plot of the duration of treatment and response as data cutoff in patients that were evaluable for antitumor activity (n=10). Subject ID and dose level are indicated. DL: Dose level. CR: Complete Response. SD: Stable Disease. PD: Progression of Disease.

All 12 patients were evaluable for adverse events. An overview of general safety is shown in Table 2, including treatment-related adverse events (TRAE). The most common TRAE were grade 1 fever, chills, and injection-site reactions, predominantly observed at DL3 and DL4 (Extended Table 2). In total, five patients experienced fever, with four of them having repetitive episodes shortly after IT injections lasting less than an hour. Four serious adverse events occurred; none of them considered to be TRAE. All TRAE were mild to moderate (grade 1 or 2), and no dose-limiting toxicities (DLTs) observed. In terms of toxicities of interest, none of the patients experienced significant decreases in platelet counts or increase in liver enzymes (Fig. 2a-c). The MTD was not reached, indicating overall safety and tolerability across all DLs. Therefore, the recommended phase 2 dose (RP2D) was established at DL4, equivalent to 10 mg.

2141-V11 demonstrated dose-proportional serum concentrations over time. Serum levels were below the limit of quantification (50 ng/mL) at DL1 and DL2, and only one out of three patients at DL3 had detectable serum levels of 2141-V11 during cycle 1. At DL4, all patients had detectable serum concentrations of 2141-V11, with the mean peak concentrations (C_max_) of 330.6 ng/mL and a time to peak concentration (T_max_) of 0.27 h (range=0.25–0.27h) (See extended Table 3). Antidrug antibodies (ADAs) were also analyzed to evaluate the potential immunogenicity of 2141-V11. ADAs were detected in four patients (DL12 n=1; DL3 n=2; DL4 n=4); two of them with the minimum required dilution (MRD) of 20-fold. The other two patients displayed dilution greater than the MRD and corresponded to patients who received the highest number of treatment cycles (Subject 06 DL2, Subject 09 DL3) (Extended Data Table 4).

### Efficacy and exploratory biomarkers of activity

Out of the 12 patients treated with 2141-V11, 10 were evaluable for antitumor activity and two had clinical progression before tumor assessment. A decrease in tumor burden was observed in six patients across all DL (DL1, n=1; DL2, n=2; DL3, n=2; DL4, n=1). Complete responses (CR) were observed in two patients: one with melanoma (DL2) and the other with hormone positive breast cancer (DL4). Stable disease (SD) was seen in six patients and progression of the disease (PD) was seen in two patients (Fig. 2d). The confirmed overall response rate (ORR) among evaluable patients was 20% (2/10), with a disease control rate (DCR) of 80% (8/10).

**Extended data Fig. 2:**
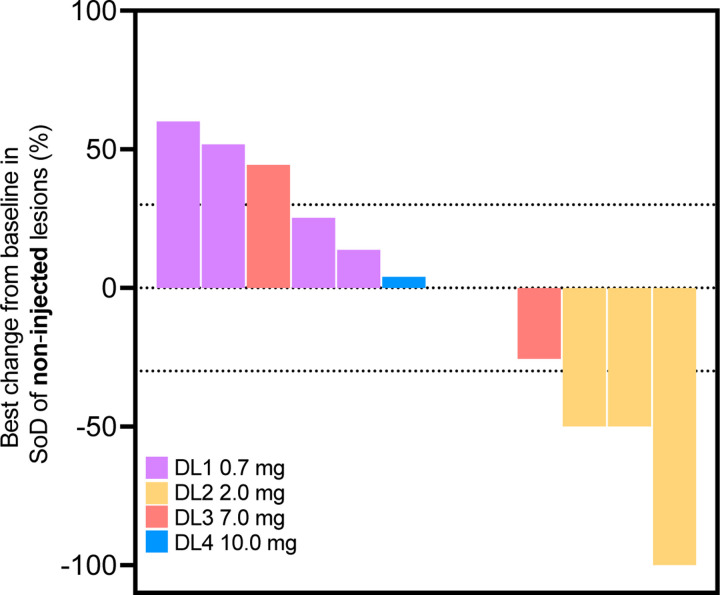
Preliminary clinical activity of 2141-V11 in non-injected lesions. Waterfall plot displaying best percent change from baseline in sum of lesion diameters (SoD) of non-injected target lesions across different dose levels (0.7 mg violate bars; 2.0 mg yellow bars; 7.0 mg salmon bars; 10 mg blue bars).

The preliminary clinical activity of 2141-V11 was further evaluated among injected and non-injected lesions^22^. Overall, a decrease in tumor burden of target lesion was observed in nine of 14 (64.2%) injected lesions. The maximal tumor shrinkage in injected target lesions ranged from 3.3% to 100%, with five achieving CR (4/14) or partial response (PR) (1/14). The injected response rate (IRR) was 35.7% (5/14) (Fig. 2e). Out of the 12 non-injected target lesions, four (33.3%) showed tumor size reduction, with maximal tumor shrinkage ranging from 25% to 100%. Three non-injected lesions achieved CR (1/12) or PR (2/12), resulting in a non-injected response rate (nIRR) of 25% (3/12; 95%) (Extended data Fig. 2). The median duration of treatment was 2.5 months (range 0.7–10.6 months). Among patients with CR, the median time of response was 3 months (Range 1.76–4.2 months), and the median duration of response (DOR) was 12 months (range 7.1–16.76 months) (Fig. 2f-2g).

**Extended data Fig. 3:**
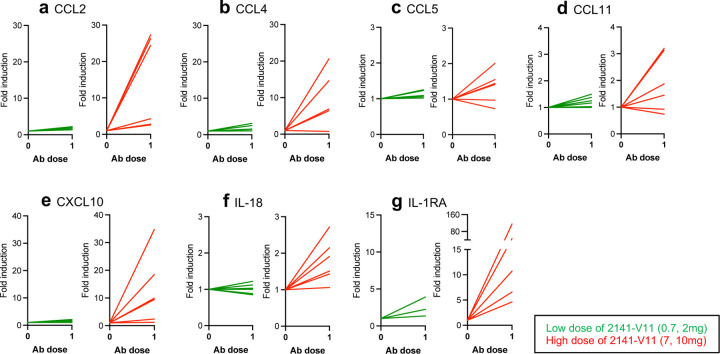
2141-V11 administration leads to systemic immune activation. **a-g** Serum levels of seven out of 34 analytes obtained from 2141-V11 trial participants pre-treatment and 24 h after the first dose of 2141-V11 treatment. All panels show the fold induction from baseline (pre-treatment) of specific analytes, per patient; Left (green) shows data for six patients who received the two lower doses of 2141-V11 (0.7 and 2mg), and right (red) shows data for six patients who received the two higher doses of 2141-V11 (7 and 10mg). Analyte levels were determined using the ProcartaPlex^™^ Human Cytokine & Chemokine Panel.

To assess whether 2141-V11 leads to systemic immune activation, peripheral blood samples were collected from all participants at pre-treatment and 24 hours after the first dose of 2141-V11. Serum analysis of a 34-panel of human cytokine and chemokines did not reveal significant differences between responding (CR) and non-responding (SD and PD) patients (data not shown). However, comparison of low (DL1 and DL2) to high dosing (DL3 and DL4) of 2141-V11 showed dose-dependent increase in levels of cytokines and chemokines associated with inflammation, immune cell activation and IFNγ signaling (CCL2, CCL4, CCL11, CCL11, CXCL10, IL-18 and IL-1RA) in the high dose group following treatment with 2141-V11 (Extended data Fig. 3).

### Clinical characterization of patients with complete responses to 2141-V11

**Figure 3.**
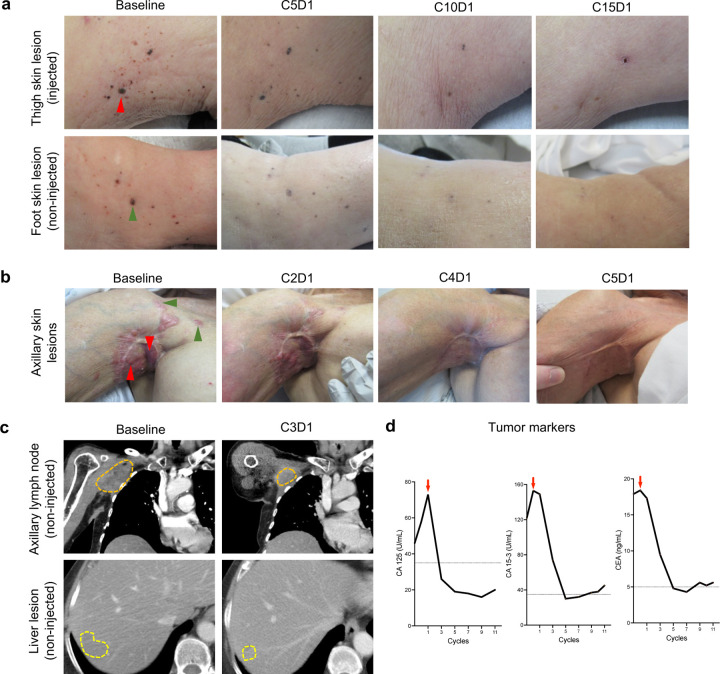
Clinical characterization of complete responders to 2141-V11. **a,** Representative photographs of the cutaneous lesions of patient 06 that achieve CR to 2141-V11. Images of injected (red arrows) and non-injected target lesions (green arrows) were obtained at baseline and at indicated cycle and day timepoints. **b,** Representative photographs of patient 14 that achieve complete response to 2141-V11. Images of injected (red arrows) and non-injected target lesions (green arrows) were obtained at baseline and at indicated post-treatment timepoints. **c,** Radiographic assessment of non-target axillary lymph nodes and liver metastases were obtained at baseline and after treatment with 2141-V11 in patient 14. **d,** Tumor markers of patient 14 before and after treatment with 2141-V11, red arrows indicate time of treatment initiation. Dashed lines indicate normal values of each tumor marker. C: Cycle. D: Day.

We further characterized the clinical course of the two patients that showed significant responses to 2141-V1. The first patient with CR was an 89-year-old woman with recurrent in-transit malignant melanoma (Stage IIIC, Subject 06). The patient was initially diagnosed ten years prior with Stage IIC melanoma for which she underwent wide local excision and sentinel LN biopsy. At the time of enrollment, patient had declined standard therapies including Talimogene laherparepec (TVEC) and systemic anti-PD-1 immunotherapy. Their ECOG status was 1, and no other major co-morbid conditions were present. Physical examination revealed multiple in-transit melanoma lesions in the proximal left thigh and left foot (Fig. 3a, baseline). Patient was enrolled in DL2 (2.0 mg) and 2141-V11 was injected into a single lesion in the left thigh. By cycle 5 of therapy, many satellite lesions began to flatten out or disappear, including non-injected lesions in both the left thigh and distal left foot. These changes became more prominent by cycle 10. At cycle 15, no notable lesions remained for injection (Fig. 3a).

The second patient with CR was a 67-year-old woman with stage IV ER(+)/PR(+)/HER-2(−) breast cancer with metastases to the liver, lymph node and soft tissue that was initially diagnosed 20 years ago (Subject 14). Past treatments included multiple rounds of surgery, radiation, and systemic chemotherapy (14 prior lines of therapy, none included systemic immunotherapy). At the time of enrollment, ECOG status was 1, and metastatic disease included cutaneous lesions in the right arm, axilla, and shoulder, with limited range of motion and decreased sensation in the right upper extremity. Patient was enrolled in DL4 (10.0 mg) and 2141-V11 was given as a single injection into the right axilla at cycle 1, and subsequently as a split dose (5 mg each) in the two most involved areas (Fig. 3b). By cycle 2, there was a measurable decrease in the cutaneous disease, with significant flattening of all target lesions (both injected and non-injected). By cycles 4 and 5 of therapy, all target lesions in the skin (injected and non-injected) had resolved (Fig. 3b). At baseline, the patient also had non-target axillary and liver metastases (Fig. 3c), and an interim CT scan before cycle 4 demonstrated decrease in size of both axillary (4.0 × 3.2 cm, previously 4.7 × 4.2 cm) and hepatic metastasis (segment 7 lesion: 0.9 × 0.6 cm, previously 1.0 × 0.9 cm, and segment 6 lesion: 0.8 × 0.8 cm, previously 1.2 × 0.8 cm) (Fig. 3c). Moreover, by cycle 4, 2141-V11 also led to normalization of all tumor markers serum levels (CA 15–3, CA-125, CEA) (Fig. 3d).

### Responses to 2141-V11 correlate with the presence of tertiary lymphoid structures (TLS) and lymphocyte infiltration in the tumor

**Figure 4.**
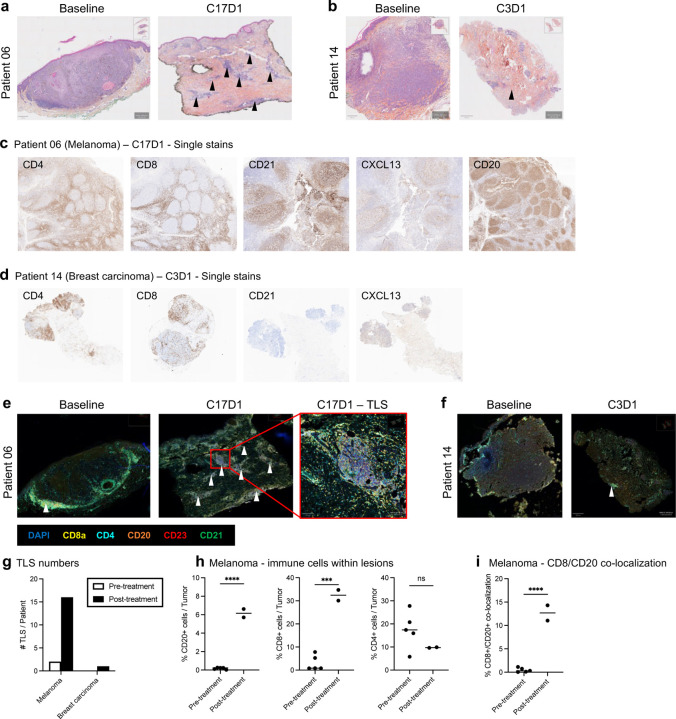
Induction of tertiary lymphoid structures (TLS) and enhanced intratumoral CD8 T-cell infiltration in complete responders to 2141-V11. **a-b,** Representative images of H&E-stained slides of tumor samples from patient 06 (**a**) and patient 14 (**b**), that were collected at baseline (left) and at the indicated post-treatment cycle (right). **c-d**, Immunohistochemistry of FFPE sections from patient 06 with melanoma after cycle 17 of 2141-V11 (**c**) and patient 14 with breast carcinoma after cycle 3 of 2141-V11 (**d**). **e-f**, Representative images of multiplex immunofluorescence (mIF) from patient 06 (**e**) and patient 14 (**f**) collected at the indicated timepoints. Tertiary lymphoid structures (TLS) are indicated (white arrows). The mIF panel consisting of the indicated markers (CD4/CD8/CD20/CD21/CD23/DAPI) was used for the characterization and quantification of TLS. **g,** Number of TLS in pre- and post-treatment biopsies from patient 06 (melanoma) and patient 14 (breast carcinoma). **h,** Infiltration of immune sub-populations (CD20+ B-cells, CD8+ T-cells and CD4+ T-cells) into the pre- and post-treatment lesions of patient 06 (melanoma). **i,** Co-localization of CD8+ and CD20+ cells within the pre- and post-treatment lesions of patient 06 (melanoma).

Confirmatory biopsies on both responding patients were performed to assess pathologic response (Fig. 4a-4b). In patient 06, residual, non-injected melanotic areas of known prior disease were shown to be free of disease, with some melanocytic pigment remaining. Of note, H&E staining revealed multiple immune infiltrates which were not present in archival baseline biopsies (Fig. 4a). IHC staining demonstrated that these immune infiltrates displayed robust infiltration of both CD8^+^ and CD4^+^ T-cells surrounding open, follicular-appearing areas comprised of CD20^+^ B-cells with high expression of the B and T follicular helper cell chemoattractant CXCL13, as well as presence CD21, which is highly expressed on follicular dendritic cells and mature B-cells. These findings were consistent with the presence of ectopic lymphoid organs known as tertiary lymphoid structures (TLS)^22^ (Fig. 4c). Multiplex immunofluorescence (IF) confirmed the increased number of TLS in the post-treatment biopsy (Fig. 4e, 4g), displaying positivity for markers of maturity including CD21 and CD23 (Fig. 4e).

**Extended data Fig. 4:**
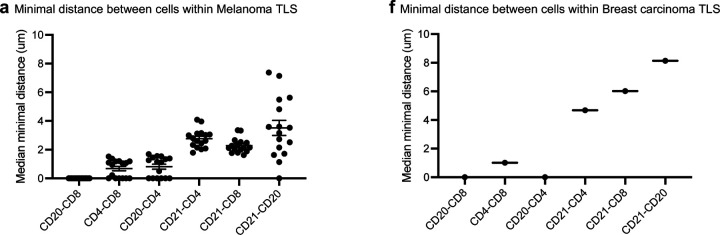
Distance analysis from multiplex immunofluorescence of individual TLS markers following 2141-V11 administration in patients with CR. **a-b.** Punch biopsies were collected from 2141-V11 trial participants after administration of 2141-V11 treatment. Multiplex immunohistofluorescence was performed on FFPE sections (CD8a/CD4/CD20/CD21/CD23/DAPI) and TLS numbers were determined. Median minimal distance between immune cells within post-treatment TLS of patient 06 (**a**) and patient 14 (**b**). Each dot represents a TLS.

In patient 14, no visible target lesions were observed after the fourth treatment cycle with 2141-V11. Biopsies from the remaining axillary soft tissue demonstrated residual breast adenocarcinoma by ER staining. Interestingly, H&E and IHC staining demonstrated an immune infiltrate positive for CD4^+^, CD8^+^ T-cells, and CD20^+^ B-cells, as well as positivity for CXCL13 and CD21, which were consistent with TLS and not present on pre-treatment biopsies (Fig. 4b, 4d). Further evaluation with multiplex IF confirmed the presence of one mature TLS, with positive staining for CD21 and CD23 (Fig. 4f-4g). Spatial co-localization analysis within the TLS of both melanoma and breast cancer post-treatment biopsies revealed that the closest interactions were observed between CD4^+^ and CD8^+^; CD20^+^ and CD8_+_ cells, and CD20^+^ and CD4^+^ cells (Extended data Fig. 4a-4b).

Multiplex IF was also used to compare the immune landscape in pre- and post-treatment biopsies from patient 06. A significant increase in the amount of CD20^+^ cells, CD8^+^ cells but not CD4^+^ cells was observed in the post-treatment samples (Fig. 4h). Moreover, the level of co-localization between CD8 and CD20 cells was substantially higher in the post-treated biopsies (Fig. 4i), suggesting that 2141-V11 promotes interactions among these two immune cell populations.

### Intratumoral 2141-V11 leads to sustained antitumor immunity and abscopal effect in an immunocompetent mouse model humanized for CD40 and FcγRs

**Extended data Fig. 5:**
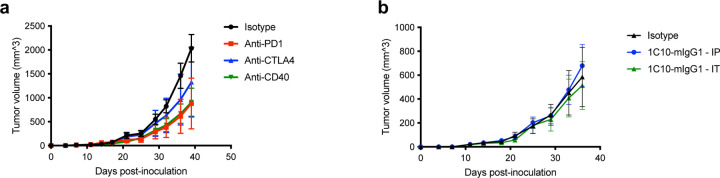
Characterizing E0771 tumors in vivo. **a,** WT C57BL/6 mice were inoculated to the mammary fat pad with 5*10^5^ E0771 tumor cells. Checkpoint modulating Abs (anti-mPD1 – clone RMP1–14 – 200ug/dose; anti-mCTLA4 – clone 9H10 – 200ug/dose; anti-mCD40 – clone 1C10 – 50ug/mouse) or isotype-matched control Abs were administered systemically, IP, on days 14, 17, 21 and 24. **b** WT C57BL/6 mice were inoculated to the mammary fat pad with 5*10^5^ E0771 tumor cells. Anti-mCD40 Abs were administered systemically (IP, as described above, in **a**) or intra-tumorally (IT), on days 14, 17, 21 and 24. Isotype-matched control Abs were administered as control. n=6–7/group. For all panels, average sizes of primary tumors ± SEM are presented in mm^3^, measured bi-weekly by caliper.

Upon observing the antitumor responses and immune activation in patients who had CR to 2141-V11, we further investigated the antitumor effects and mechanisms of action of CD40-targeting Abs using mouse models. In the current trial, the two patients with CR had melanoma and breast carcinoma. We have previously described the response of IT administration of 2141-V11 in a B16 melanoma model^23^. Therefore, in this project, we focus our studies on an aggressive breast cancer model syngeneic to C57BL/6 utilizing orthotopic implantation of E0771 tumor cells. In WT mice, E0771 tumors failed to respond to systemic treatment with Abs targeting immune checkpoints such as PD1 and CTLA-4 (Extended data Fig. 5a). Furthermore, systemic or IT administration of an anti-mouse CD40 Ab (Clone 1C10-mIgG1) failed to induce tumor regression (Extended data Fig. 5b), suggesting that to achieve optimal antitumor activity, anti-CD40 Abs require Fc-engineering to enhance their interaction with the human receptor FcγRIIB and potentiate down-stream signaling. To overcome the limitations of WT mice, we utilized hCD40/hFcγR mice^15^, a strain that allows investigating the activity of human IgG Abs against human CD40 designed for the clinic, in the context of human Fc-FcγRs interactions.

**Figure 5.**
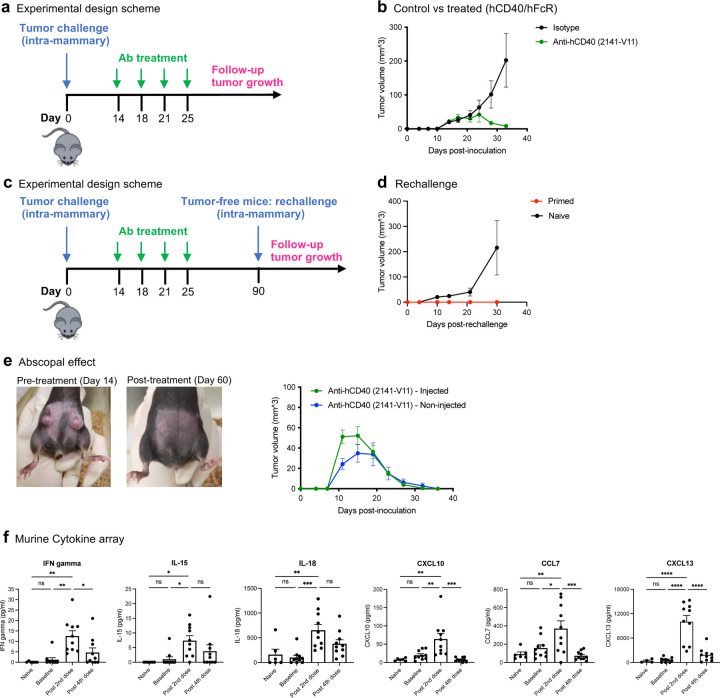
Intratumoral administration of 2141-V11 leads to antitumor immunity, resulting in primary tumor regression, an abscopal effect, and induction of long-term immune memory. **a,** Scheme of experimental design of experiment shown in (b): hCD40/hFcγR mice were inoculated to the mammary fat-pad with 5*10^5^ E0771 tumor cells. On day 14, 10 ug of anti-hCD40 Abs (2141-V11) or isotype-matched control Abs were administered IT on days 14, 17, 21 and 24. **b,** Average sizes of primary tumors ± SEM are presented in mm^3^, measured bi-weekly by caliper. n=12/group. **c,** Scheme of experimental design of experiment shown in (d): Primary tumor challenge and treatment were conducted as described above for (a-b). On day 90, mice that had cleared the tumors (termed herein “Primed” mice) and Naïve hCD40/hFcγR mice were rechallenged with 5x dose of E0771 tumor cells (2.5*10^6^ cells/mouse). **d,** Average sizes of primary tumors ± SEM are presented in mm^3^, measured bi-weekly by caliper. n=9–12/group. **e,** Primary tumor challenge and treatment were conducted as described above for (a-b), but tumor cells were inoculated into two mammary fat pads. For each mouse, the antibodies were administered only into a single tumor, the largest of the two. Images are from the same mouse on day 14 (prior to treatment initiation) and day 60. n=12mice/group. **f,** Mice were challenged and treated as described above for (a-b). Blood was collected three times: on day 14 pre-treatment (“baseline”), 24 h after the second and fourth doses of the Ab treatment. Blood from Naïve (tumor-free) mice served as control. Serum levels of 36 analytes were determined using the ProcartaPlex^™^ Mouse Cytokine & Chemokine Panel. mCXCL13 was detected using the R&D CXCL13 Duoset ELISA kit. n=10/group (naïve mice = 6mice/group). * p<0.05, ** p<0.01, *** p<0.001.

hCD40/hFcγR mice were inoculated orthotopically with E0771 tumor cells. Starting on day 14, mice were treated biweekly with IT injections of 2141-V11, or isotype-matched control (Fig. 5a). In contrast to experiments performed in WT mice, treatment with 2141-V11 led to complete regression of tumors by day 35 in hCD40/hFcγR mice (Fig. 5b). Considering the durable, long-term responses observed in the two patients with CR (Fig. 2f), we investigated whether 2141-V11 could also induce long-term immune memory in these mice, protecting them from disease recurrence (Fig. 5c). Ninety days after tumor implantation, we rechallenged the mice that achieved CR following 2141-V11 therapy (Primed mice), with five times the initial dose of E0771. We observed that, unlike control mice (Naïve mice), the Primed mice were protected from tumor rechallenge, with tumors failing to engraft (Fig. 5d), indicating that 2141-V11 leads to long-term immunity against E0771 tumors.

In our clinical trial, we also observed regression of non-injected lesions (Extended data Fig. 2). This observation prompted us to investigate whether this abscopal effect also occurs in mice. We have previously demonstrated that in the aggressive B16 melanoma model, 2141-V11 alone failed to elicit an abscopal effect and required combination therapy with anti-PD1 therapy^23^. To address this question in the breast cancer model, we used a bilateral E0771 breast tumor model which allows monitoring the growth of injected and non-injected tumors. Treatment with 2141-V11 led to rejection of both injected and non-injected tumors (Fig. 5e), suggesting that the CD40-targeting Ab led to a systemic and robust immune activation, extending beyond the local tumor.

### 2141-V11 leads to local and systemic immune activation in vivo

Similar to the peripheral cytokine profile performed in patients (Extended data Fig. 3), we analyzed the cytokine profile of E0771 tumor-bearing mice treated with 2141-V11. We observed that serum levels of several cytokines including IFN-gamma, IL-15, IL-18, CXCL10, CCL7, and CXCL13 were significantly upregulated after the second dose but returned to baseline after the fourth dose (Fig. 5f). Similarly, we observed CXCL13 expression in the lesions of patients with CR (Fig. 4c-4d). Notably at pre-treatment (at “Baseline”), all cytokine levels were comparable to the cytokine levels of tumor-free mice (Naïve), suggesting that the 2141-V11 Ab led to this systemic immune activation, and not the mere presence of the tumor (Fig. 5f).

**Figure 6.**
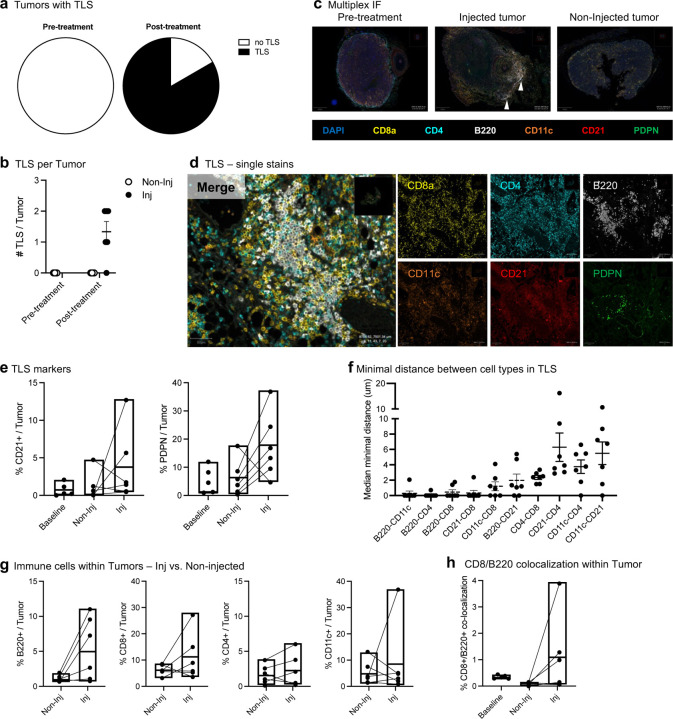
Intratumoral administration of 2141-V11 leads to TLS formation within the injected tumors. **a-h,** hCD40/hFcγR mice were inoculated into two mammary fat pads with 5*10^5^ E0771 tumor cells. On day 14, 10 ug of anti-hCD40 Abs (2141-V11) were administered intra-tumorally on days 14 and 17. For each mouse, the antibodies were administered only into a single tumor, the largest of the two. Tumors were excised on day 14 (baseline, pre-treatment) or on day 18 (24 h after the second Ab dose). FFPE tumor sections were stained for CD8a, CD4, CD11c, B220, CD21, and Podoplanin (PDPN), and TLS numbers were determined. n=6/group. **a,** Number of tumor samples that contained any number of TLS. **b,** Quantitation of TLS numbers per tumor sample. **c,** Images of representative tumors: pre-treatment (baseline), and post-treatment - injected and non-injected tumors. **c,** Zoomed-in image of TLS structures, from a baseline tumor and an injected tumor. **d,** Single stains of immune markers within a TLS from an injected tumor. **e,** Quantitation of TLS markers – CD21 and PDPN. **f,** Median minimal distance between immune cell types within post-treatment TLS in injected tumors. **g,** Infiltration of immune cell types (B220+ B-cells, CD8+ T-cells, and CD4+ T-cells) into the injected and non-injected tumors. **h,** Co-localization of CD8+ and CD20+ cells within the pre- and post-treatment tumors.

Given the observation that treatment with 2141-V11 was associated with the presence of TLS in post-treatment biopsies (Fig. 4), we investigated whether CD40 agonism with 2141-V11 would induce the formation of TLS in injected and non-injected tumors using the bilateral E0771 breast tumor model. Multiplex IF staining (B220/CD8a/CD4/CD11c/CD21/Podoplanin) demonstrated that at baseline, no TLS were observed in any of the implanted tumors (0/6 baseline tumors; Fig. 6a, 6c). In the post-treatment samples, TLS were observed only in injected tumors with 2141-V11 (5/6); none of the non-injected tumors had presence of TLS (0/6) (Fig. 6a-6c). Further characterization of the TLS revealed large B220^+^ B-cell centers surrounded by numerous CD4^+^ and CD8^+^ T-cells and some CD11c^+^ DCs (Fig. 6d). Quantification of CD21 and podopolanin were substantially elevated only in injected tumors, supporting the presence of TLS (Fig. 6e). Spatial co-localization analysis within the TLS of injected tumors revealed that the closest interactions observed were between B-cells and CD11c^+^, CD8^+^, and CD4^+^ T-cells, and also between CD11c^+^ and CD8^+^ cells (Fig. 6f).

As 2141-V11 led to an abscopal effect in the bilateral E0771 tumor model, we compared the immune landscape between injected and non-injected tumors. We observed that injected tumors displayed increased numbers of CD8^+^ and B-cells as compared to non-injected tumors, and similar levels of CD4^+^ and CD11c^+^ cells (Fig. 6g). Moreover, co-localization between CD20^+^ B-cells and CD8^+^ T-cells was substantially higher in injected tumors (Fig. 6h), suggesting that local administration of 2141-V11 promotes immune synapses among these cell populations.

## Discussion

Several agonistic CD40-targeting Abs have been evaluated in clinical trials over the last two decades. However, none of these Abs have advanced beyond early clinical trial phases, particularly due to high toxicity (liver toxicity and thrombocytopenia) and limited antitumor activity^8–14^. In this study, we investigated the safety and preliminary clinical activity of IT administration of 2141-V11. Our findings show that IT 2141-V11 is safe and induces effective antitumor responses in a subset of patients. All TRAE were mild (grade 1–2), and the MTD was not reached. Based on activity, tolerability, and pharmacokinetic profile the RP2D was determined to be 10 mg for IT administration. The ORR and DCR were 20% and 80%, respectively, with two patients achieving CR. Importantly, antitumor activity was observed in tumors traditionally responsive to immunotherapy, such as melanoma, and in less responsive ones, such as breast cancer. Our results indicate that the relatively low clinical response observed in past clinical studies may be attributable to the type of Ab used, specifically their Fc portion, which is not optimized for strong binding to FcγRIIB. Furthermore, we also found that administering the Ab directly into the tumor site circumvents the toxicity associated with systemic administration of anti-CD40 Abs and directs their activity to the tumor microenvironment. Importantly, prior intralesional injection of a parental IgG1 anti-CD40 Ab was demonstrated safe when administered intralesionally^11^; however, it had no demonstrable anti-tumor effects, further supporting the importance of the Fc portion for optimal in vivo activity.

Consistent with our earlier studies in animal models^15–18^, in post-treatment biopsies from patients, we observed that 2141-V11 was associated with increased CD8^+^ T-cell infiltration and enhanced interactions with CD11c^+^ or B-cells, which may act as APCs. These changes in the TME resulted in improved antitumor immunity locally and systemically, as indicated by upregulation of multiple cytokines, regression of remote metastases in patients, and abscopal antitumor effects observed in hCD40/hFcγR mice. Moreover, 2141-V11 facilitated the development of long-term immune memory in mice, which might explain the sustained responses observed in responding patients. These results confirm the ability of CD40 stimulation to promote T-cell responses and effective antitumor immunity.

We also found that treatment with 2141-V11 in the patients with CR promoted the formation of TLS at the non-injected sites. TLS are ectopic lymphoid organs composed of immune (T-cells, DC and B-cells), and non-immune cells (high endothelial venules, and stromal cells) that have been recently proposed as privileged sites for immune cell infiltration, tumor antigen presentation, and activation/proliferation of CD8^+^ T-cell and B-cells^24–26^. Several studies have associated the presence of TLS with better prognosis across various malignancies^27^, and with improved responses to immunotherapies^28^. However, there is limited data on which pathways mediate *de novo* induction of TLS by immunotherapies^29,30^. Moreover, modeling TLS induction in mice has proved challenging and required complex strategies such as artificial scaffolds^29^ or implantation of engineered stromal cell lines^30^. Our studies demonstrate the induction of TLS by CD40-targeting Abs in our humanized mouse system^31^, serving as a valuable platform to study the development and function of immunotherapy-induced TLS.

The current clinical study is limited by its small sample size and by the limited number of participants with a particular tumor type, which makes it difficult to predict which cancer type is most likely to respond to CD40-agonistic Abs. However, the encouraging safety profile of 2141-V11, and the profound clinical responses observed in a patient typically unresponsive to immunotherapies, open opportunities for further investigation in specific tumor types in phase II studies, either as monotherapy or in combination with other complementary immunotherapies.

In summary, our study demonstrates that IT administration of 2141-V11 is a safe and potentially effective therapeutic approach in a subset of patients with cancer. Notably, in patients responding to 2141-V11, the antitumor effects are systemic, profound, and durable, justifying further phase II studies of 2141-V11 in specific tumor types. Our ability to use preclinical models that recapitulate the human expression of CD40 and FcγRs has allowed us to prioritize the most biological rational approaches and which tumor types to focus on in future studies with 2141-V11, either alone or in combination with standard or novel therapies. Understanding the mechanisms by which CD40-targeted Abs drive effective antitumor immunity and associated toxicity provide the rationale on how to improve this promising therapeutic approach.

## Methods

### NHP pharmacokinetics and toxicology

NHP studies were performed by Charles River Laboratories (France Safety Assessment SAS) in accordance with the OECD Principles of Good Laboratory Practice accepted by Regulatory Authorities throughout the European Union, United States of America (FDA and EPA), and Japan (MHLW, MAFF and METI). In summary, a total of 32 Cynomolgus monkeys were dosed in four groups: Group 1: Control IV/SC (n=10); Group 2: 2141-V11 at 3 mg/Kg/dose SC (n=6); Group 3: 2141-V11 at 3 mg/Kg/dose IV (n=6); Group 4: 2141-V11 at 100 mg/Kg/dose SC (n=10). The following parameters and end points were evaluated in this study: clinical observations, body weights, electro-cardiology, clinical pathology parameters (hematology, coagulation, clinical chemistry, and urinalysis), bioanalysis, toxicokinetic parameters, anti-therapeutic antibody analysis, immunophenotyping, gross necropsy findings, organ weights, and histopathologic examinations. Blood was collected from the femoral vein. Urine was collected from animals housed in individual cages overnight, deprived of food but with access to water. The pre-treatment repeat urine sample was collected over a period of approximately one hour, during which the animal was singly housed and deprived of food but had access to water. Animals were deprived of food before blood sampling.

### Study design and participants.

This was a single-center, phase 1, open-label study (NCT04059588) to assess the safety and tolerability of the Fc-engineered variant 2141-V11 in patients with solid tumors locally advanced or metastatic to the skin amenable to intratumoral injection. The study included only a dose-exploration phase. A traditional 3 + 3 dose escalation design was used, with up to three participants enrolled at doses of 0.7, 2.0, 7.0, and 10.0 mg to determine the MTD. 2141-V11 was administered once every three weeks, on day 1 of a 3-week cycle. MTD was defined as 1 dose level below the dose in which DLTs are observed in > or = 33% of the participants.

At screening, patients were eligible if they were aged ≥18 years; had an Eastern Cooperative Oncology Group performance status of 0–1; had confirmed refractory or relapsed disease with measurable or evaluable metastatic disease (at least more than 1 lesion) as evidenced by physical exam or imaging; had an identifiable metastatic lesion of the skin, subcutaneous tissue, or lymph node amenable to intratumoral injection; had exhausted or declined standard-of-care therapy for their disease; had at least 4 weeks since treatment with immune checkpoint inhibitors or other antibody-based therapy or investigational agents, and at least 2 weeks since chemotherapy, targeted small molecule therapy, cytokine therapy, or radiation therapy. Patients with abnormal blood counts or liver enzymes, history of pneumonitis, active hepatitis B, autoimmune disease, clinically active brain or leptomeningeal disease, treatment with immunosuppressive regiments within 30 days prior to enrollment, stroke, or intracranial hemorrhage within six months prior to enrollment, vaccination with live attenuated viral vaccines, major surgery, and severe infection or hospitalization within four weeks of enrollment were excluded from the study.

The study was conducted in accordance with the Declaration of Helsinki and International Council Harmonization Good Clinical Practices Guidelines. The study protocol, amendments, and informed consent were approved by the institutional review board. The study complied with local regulation governing the conduct of clinical studies and institutional guidelines. All patients provided written, informed consent. The data were collected by the sponsor and all authors had full access and were involved in data interpretation, manuscript preparation, revision, and final approval. The authors vouch for the accuracy of the data and adherence to the study protocol.

### Intratumoral injections:

2141-V11 was administered by a qualified licensed healthcare professional (e.g., an oncologist). Subjects were assessed per schedule of events with laboratory results confirmed before each treatment. Dosing occurred only if these test values are acceptable. 2141-V11 was administered by intralesional injection only into injectable cutaneous or subcutaneous tumors or involved lymph nodes. The first cycle of 2141-V11 was 21 (+3) days. Subsequent cycles were given every 21 (±3) days at the same dose level. Dose reduction for adverse events were not allowed. Patients remained on treatment until they have achieved complete response; all injectable tumors have disappeared; there was confirmed disease progression; unacceptable toxicity; or consent withdrawal.

### Assessments

Incidence and nature of adverse events was assessed for severity using Common Terminology Criteria for Adverse Events (CTCAE) v.5.0. Changes in vital signs, physical findings, and clinical laboratory results during and following 2141-V11 administration were monitored. PK assessments and incidence of ADA responses to 2141-V11 were performed in all patients. Preliminary clinical activity was assessed by treating physician in accordance with Response Criteria for Intratumoral Immunotherapy in Solid Tumor itRECIST^22^ and RECIST v1.1 criteria, using measurement of target and non-target lesions by caliper and/or computed tomography (CT) as clinically indicated.

### Endpoints:

The primary endpoints were to determine the safety and tolerability of intratumoral 2141-V11, and to determine the MTD and RP2D of 2141-V11 in patients with cancer. The secondary endpoints were to determine the pharmacokinetic and safety measures of 2141-V11 in participants with cancer, including characterization of the PK (C_max_, C_trough_ where PK data was available, AUC, terminal and effective half-lives, etc.) as well as determine preliminary clinical activity of 2141-V11 (determined as best overall response, overall response rate, duration of response, disease control rate, injected and non-injected response rate, and progression-free survival). Exploratory objectives including understanding relevant markers in circulation and tissue as indicators of the immune-modulator effect and anti-tumor activity of 2141-V11.

### Antibody engineering and production

The Fc portion of hIgG1 agonistic anti-hCD40 antibodies (parental clone: CP-870,893) was Fc-engineered to enhance binding for the inhibitory FcγRIIB by introducing five-point mutations G237D/P238D/H268D/P271G/A330R (termed herein “2141-V11”), as previously described^15^. Production of 2141-V11 for clinical use under GMP conditions was performed by MassBiologics. It was produced by recombinant cell cultures, purified, and sterilized. The 2141-V11 drug product is formulated as a 20 mg/mL solution in 10 mM Histidine; 150 mM sodium chloride; 0.025% Polysorbate 80 at pH 6.0. The high concentration was maintained to allow maximal drug delivery in minimal volume for intralesional delivery.

The variable heavy and light regions of anti-mCD40 antibody (clone 1C10), anti-mPD-1 (clone RMP1–14), and anti-mCTLA4 (clone 9H10) were synthesized (IDT) and cloned into mammalian expression vectors with mouse IgG heavy chains and mouse kappa light chains, as previously described^16^. For the generation of an Fc-null variant of mouse IgG1 (D265A), site-directed mutagenesis using specific primers was performed based on the QuikChange site-directed mutagenesis Kit II (Agilent Technologies) according to the manufacturer’s instructions. Mutated plasmid sequences were validated by direct sequencing (Genewiz). Antibodies were generated by transient co-transfection with a heavy chain and light chain constructs of Expi293F cells. Expi293F cells were maintained in serum-free Expi293 Expression Medium and transfected using ExpiFectamine^™^ 293 Transfection Kit (All from Thermo Fischer Scientific). Supernatants were collected seven days post-transection, centrifuged, and filtered (0.22 mm) to eliminate any remaining cells. Antibodies were purified from the supernatants using Protein G Sepharose 4 Fast Flow (GE Healthcare), dialyzed in PBS, and sterile filtered (0.22 mm) as previously described^32^. Isotype control antibodies (hIgG1 and mIgG) directed against irrelevant viral proteins were generated, as described above, to serve as control in murine experiments.

### Mice

Humanized mice containing human Fc receptors (FcgRa^null^, hFcgRI+, FcgRIIa^R131+^, FcgRIIb^+^, FcgRIIIa^F158+^, and FcgRIIIb^+^) and human CD40 (termed herein “hCD40/hFcγR”) were generated and extensively characterized as previously described^15^. WT C57BL/6 mice were purchased from The Jackson Laboratories. All mice were maintained in The Rockefeller University Comparative Bioscience Center. All experiments were performed in compliance with institutional guidelines and had been approved by The Rockefeller University Institutional Animal Care and Use Committee (IACUC).

### In vivo tumor models and treatment

E0771 (5*10^5^ cells/mouse) cells were inoculated orthotopically into the fourth mammary fat pad in 100ul PBS. Tumor volumes were measured bi-weekly with an electronic caliper and reported as volume (mm^3^) using the formula (L1^2^*L2)/2, where L1 is the shortest diameter and L2 is the longest diameter. On day 14, mice were randomized and received intratumoral (or intraperitoneal) injections of checkpoint-modulating antibodies or isotype-matched IgGs serving as control, on days 14, 17, 21 and 24 post-inoculations. The following antibodies were administered, all in 100ul PBS: anti-hCD40 (2141-hIgG1-V11; 0.2mg/kg), anti-mCD40 (1C10-mIgG1; 0.5mg/kg), anti-mPD-1 (RMP1–14-mIgG1-D265A; 10mg/kg), anti-mCTLA4 (9H10-mIgG2a; 10mg/kg).

In experiments evaluating an abscopal effect – mice were inoculated bi-laterally, into the 4^th^ mammary fat pads. For each mouse, the antibodies were administered, as described above, into the largest of the two tumors.

In experiments evaluating immune memory – on day 90, tumor-free mice that cleared primary tumors following treatment (as described above) were rechallenged with 5x dose of E0771 tumor cells (2.5*10^6^ cells/mouse) in 100 ul PBS. Naïve mice were challenged simultaneously and served as control.

### Multiplex Cytokine Analysis (mouse and human)

For patient samples – blood samples were taken before treatment initiation and 24 hours after administration of the first dose of 2141-V11. Samples were processed for plasma and stored at - 80°C until assays were performed. Plasma (25 µl) was analyzed in duplicates using human cytokine and chemokines 34-plex panel 1A (EPX340–12167, ProcartaPlex^™^, eBioscience) according to the manufacturer’s guidelines. Fluorescence was acquired on a Luminex^®^ 200^™^ System and analyzed using ProcartaPlex Analyst 1.0 software.

For murine samples – blood was collected from E0771-tumor bearing mice on day 14, before the first dose of 2141-V11 was administered (termed herein “baseline”) and then 24 h after the second and fourth doses (tumor-free mice served as control, termed herein “Naïve”). Plasma (25 µl) was analyzed in duplicates using mouse cytokine and chemokines 36-plex panel 1A (EPXR360–26092, ProcartaPlex^™^, eBioscience) according to the manufacturer’s guidelines. Fluorescence was acquired on a Luminex^®^ 200^™^ System and analyzed using ProcartaPlex Analyst 1.0 software. Serum levels of mouse CXCL13 were detected using the Duoset ELIA kit (DY470, R&D Systems), according to the manufacturer’s guidelines.

### Multiplexed IHC (mouse and human)

Punch biopsies were collected from responding patients pre-treatment and at the indicated time points. Immunohistochemistry (IHC), and multiplex Immunofluorescence (mIF) analyses were performed by Explicyte Immuno-oncology (Bordeaux, France) on the automated Ventana Discovery XT staining platform (Ventana Medical Systems). FFPE slides of tumor tissue were deparaffinized, and antigen retrieval was performed by heat-induced epitope retrieval using standard CC1 reagent (Tris-based buffer, pH 8.0; Ventana Medical Systems). The slides were then incubated with primary antibodies against the following molecules, according to panel composition: CD8 (C8/144B, Ventana), CD4 (SP35, Ventana), CD20 (L26, Ventana), CD21 (2G9, Cell Marque), CD23 (SP23, Ventana). Bound primary antibodies were detected using OmniMap HRP-conjugated antibodies, followed by tyramide signal amplification using opal fluorophores (Opal 480, Opal 520, Opal 570, Opal 620, Opal 690, and Opal 780; Akoya Biosciences) for mIF or DISCOVERY Purple or a 3,3′-diaminobenzidine (DAB) chromogen detection kit (Roche) for IHC. The slides were ultimately counterstained with either spectral 4′,6-diamidino-2-phenylindol (Akoya Biosciences) or hematoxylin (Roche), cover-slipped, and digitized using a multispectral imaging platform (Vectra Polaris). Adjacent slides were stained with H&E (Hematoxylin and eosin). TLS and tumor-immune phenotypes were defined by expert pathologists and annotated using PhenoChart (Akoya). TLS were defined as aggregates of CD20+ and CD3+ cells (composed of at least 50 cells) located within the tumor or a maximum of 1 mm from the tumor edges.

For murine samples – the process was conducted as described above, using E0771 tumors excised from hCD40/hFcγR mice on day 14 (pre-treatment) or 24 h after administration of the second dose of 2141-V11. The primary antibodies used for immunostaining: CD8 (D4W2Z, CST), CD4 (D7D2Z, CST), B220 (RA3–6B2, Biolegend), CD21 (SP186, Abcam), CD11c (D1V9Y, CST), Podoplanin (Pmab-1, Abcam).

mIF cell type annotation and minimal distance construction - CD8+ T-cells, CD4+ T-cells, CD20+ B-cells, CD21+ populations were identified based on single-marker positivity per cell in human samples. Similarly, single-positively stained cells were used to define CD8+ T-cells, CD4+ T-cells, B220+ B-cells, CD11c dendritic cells, CD21+ populations in mouse samples. Minimal distance between immune cell populations were defined by the minimal pairwise Euclidean distance between all cells from two cell populations within a slide.

### Statistical analysis

For murine experiments – Statistical analyses were performed using GraphPad Prism Software Version 10. P-values for multiple groups were calculated via one-way ANOVA with a Tukey’s post-hoc test. When two groups were compared – an unpaired two-tailed t test was used. P-values < 0.05 were considered statistically significant (*p<0.05, **p<0.01, ***p<0.001, ****p<0.0001).

## Extended Data

**Extended Table 1.** Patient characteristics, including dose levels and number of cycles

Subject #	Age	Gender	Prior Chemotherapy	Prior immunotherapy	Cancer type	Dose (mg)	# cycles
01	55	F	Yes	Yes	TNBC	0.7	2
02	75	F	Yes	No	TNBC	0.7	5
05	67	F	Yes	No	TNBC	0.7	2

06	89	F	No	No	Cutaneous melanoma	2.0	13
07	63	F	Yes	No	TNBC	2.0	4
08	57	F	Yes	Yes	TNBC	2.0	5

09	48	F	No	Yes	Cutaneous melanoma	7.0	16
10	54	M	No	Yes	ccRCC	7.0	9
12	39	M	No	Yes	ccRCC	7.0	3

14	67	F	Yes	No	ER+ Breast Cancer	10	3
11	63	F	Yes	Yes	TNBC	10	2
15	78	F	Yes	Yes	Mucosal Melanoma	10	4

**Extended Table 2a.** Any grade AE during treatment with 2141-V11 by Dose Level

Any-Grade AE n (%)	0.7 mg	2 mg	7.0 mg	10 mg
N	3	3	3	3
Fever*	0	0	3	2
Pain	1	2	2	0
Rigor/Chills*	0	0	1	2
Injection site reaction*	0	1	2	0
Infection	0	2	0	1
Fatigue	0	1	1	0
Myalgias^Φ^	0	1	1	0
Dyspnea	0	0	1	1
Anemia	0	0	2	0
Rash^Φ^	0	0	1	0
Nausea	1	0	0	0
Hot flashes^Φ^	0	1	0	0
Dysphagia	0	1	0	0
Sinus bradycardia	0	0	1	0
Depression	0	0	1	0

* Treatment related adverse event

Φ Possibly related to 2141-V11

**Extended Table 2b.** Grade 3–4 AE by Dose level.

Grade ≥3 AE n (%)	0.7 mg	2 mg	7.0 mg	10 mg
Pain	0	0	1	0
Infection	0	0	0	1
Anemia	0	0	2	0

**Extended Table 3a.** Summary of serum concentrations of 2141-V11 in cycle 1 (ng/mL)

Cycle/Day	Timepoint	Statistics	Dose 1 0.7 mg (n=3)	Dose 2 2.0 mg (n=3)	Dose 3 7.0 mg (n=3)	Dose 4 10.0 mg (n=3)
Cycle 1 - Day 1	Predose	n	3	3	3	3
		Mean	0.000	0.000	0.000	0.000
		SD	0.000	0.000	0.000	0.000
		Median	0.000	0.000	0.000	0.000
		Min	0.000	0.000	0.000	0.000
		Max	0.000	0.000	0.000	0.000
		CV%	NC	NC	NC	NC

	15 minutes	n	3	3	3	3
		Mean	0.000	0.000	45.400	564.600
		SD	0.000	0.000	78.640	611.800
		Median	0.000	0.000	0.000	362.200
		Min	0.000	0.000	0.000	79.660
		Max	0.000	0.000	136.200	1252.000
		CV%	NC	NC	173.2	108.4

	2 hour	n	3	3	3	3
		Mean	0.000	0.000	48.900	286.400
		SD	0.000	0.000	84.700	323.400
		Median	0.000	0.000	0.000	142.200
		Min	0.000	0.000	0.000	60.070
		Max	0.000	0.000	146.700	656.800
		CV%	NC	NC	173.2	112.9

	6 hour	n	3	3	3	3
		Mean	0.000	0.000	41.53	93.53
		SD	0.000	0.000	71.94	162
		Median	0.000	0.000	0.000	0.000
		Min	0.000	0.000	0.000	0.000
		Max	0.000	0.000	124.6	280.6
		CV%	NC	NC	173.2	173.2

	24 hour	n	3	3	3	3
		Mean	0.000	0.000	0.000	0.000

Cycle 1 - Day 4	96 hour	n	2	3	3	3
		Mean	0.000	0.000	0.000	0.000

Cycle 1 - Day 8	192 Hour	n	2	3	3	3
		Mean	0.000	0.000	0.000	0.000

Cycle 1 - Day 15	360 Hour	n	2	3	3	3
		Mean	0.000	0.000	0.000	0.000

CV% = Coefficient of variation; Min = Minimum; Max = Maximum; SD = Standard Deviation; NC = Not Calculable. n = number of PK evaluable patients

**Extended Table 3b.** Summary of pharmacokinetics parameters (ng/mL)

Cycle/Day	Parameter	Dose 1 0.7 mg (n=3)	Dose 2 2.0 mg (n=3)	Dose 3 7.0 mg (n=3)	Dose 4 10.0 mg (n=3)
Cycle 1	C_max_				
	n	0	0	1	3
	Geometric Mean			NC	330.6
	Geometric CV%			NC	238.9
	T_max_	0	0		
	N			1	3
	Median			NC	0.27
	Min			NC	0.25
	Max			NC	0.27

CV% = Coefficient of variation; Min = Minimum; Max = Maximum; SD = Standard Deviation; NC = Not Calculable. n = number of PK evaluable patients

**Extended Table 4.** Anti-drug antibodies levels to 2141-V11

ID	Cancer type	DL	# cycles	Cycle																	
				1	2	3	4	5	6	7	8	9	10	11	12	13	14	15	16	17	18
01	TNBC	1	2	-	-																
02	TNBC	1	5	-	-	-	-	-													
05	TNBC	1	2	-	-																

06	Mel	2	13	NA	-	-	20	160	2560	2560	2560	2560	2560	1280	1280	1280	1280	1280	640	320	160
07	TNBC	2	4	-	-	-	-														
08	TNBC	2	5	-	-	-	-	-													

09	Mel	3	16	-	160	320	640	1280	1280	1280	1280	1280	640	640	640	320	320	160	160		
10	ccRCC	3	9	-	-	-	-	20	20	20	-	-									
12	ccRCC	3	3	-	-																

14	ER+BC	3	3	-	-	-	-	20	40	20	20	20	20	20							
11	TNBC	3	2	-	-																
15	Muc Mel	3	4	-	-	-	-														

Blood was drawn before each cycle, immediately prior to administration of 2141-V11.

Anti-drug antibody levels are based on the sample dilution factor.

Mel: Melanoma; TNBC: Triple negative breast cancer, ER+BC: Estrogen receptor positive breast cancer, ccRCC: clear cell renal cell carcinoma, Muc Mel: mucosal melanoma

## Figures and Tables

**Scheme 1:**
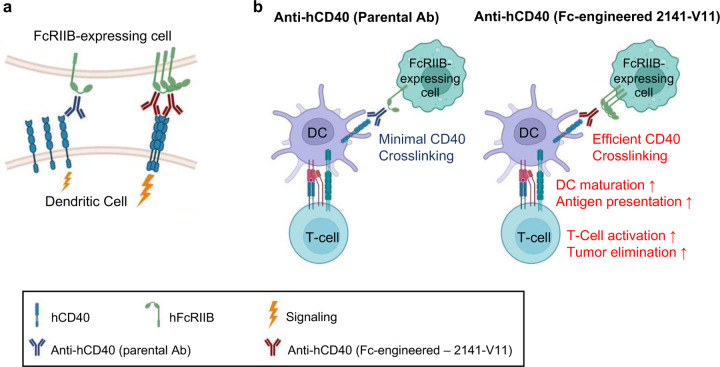
Proposed mechanism of action of anti-CD40 Abs. **a,** Fc-engineered anti-hCD40 Abs with enhanced affinity to hFcγRIIB, lead to trimerization of CD40, which results in down-stream signaling within the CD40-expressing cell. **b,** Left **–** Parental anti-hCD40 Abs are unable to induce efficient cross-linking of DC-expressed CD40. Thus, DCs do not undergo maturation and do not cross-present antigens to T-cells. Right – Fc-engineered anti-hCD40 with enhanced affinity to hFcγRIIB, lead to optimal cross-linking of CD40, trimerization which results in down-stream signaling, DC activation, efficient antigen cross-presentation to T-cells, which results in tumor elimination. Created in BiorRender.com.

**Table 1.** Demographics and baseline disease characteristics

	n=12
Age, years (min-max)	64.2 (42–89)

**Sex, n(%)**	
Female	10 (83.3)
Male	2 (16.7)

**Race, n (%)**	
White	7 (58.3)
African American	1 (8.3)
Asian	3 (25)
Other	1 (8.3)

**Baseline ECOG status, n (%)**	
0	1 (8.3)
1	11 (91.6)

**Previous palliative chemotherapy n (%)**	
Yes	8 (66.6)
No	4 (33.3)

**Previous Immune-based therapy n (%)**	
Yes	7 (58.3)
No	5 (41.6)

**Prior lines of systemic palliative therapy n (%)**	
0	1 (8.3)
1–2	1 (8.3)
≥3	10 (83.3)

**Type of solid tumor, n (%)**	
Mucosal melanoma	1 (8.3)
Cutaneous melanoma	2 (16.7)
Renal cell carcinoma	2 (16.7)
Hormone positive breast cancer	3 (25.0)
Triple negative breast cancer	4 (33.3)

**Table 2.** Any grade adverse events during treatment with 2141-V11 (n=12)

Adverse events	All grade	Grade ≥ 3
Fever*	5 (42)	0
Pain	4 (33)	1 (8)
Injection site reaction*	3 (25)	0
Rigor/Chills*	3 (25)	0
Infection	3 (25)	1 (8)
Fatigue	2 (16)	0
Myalgias^Φ^	2 (16)	0
Dyspnea	2 (16)	0
Anemia	2 (16)	2 (16)
Rash^Φ^	1 (8)	0
Nausea	1 (8)	0
Hot flashes^Φ^	1 (8)	0
Dysphagia	1 (8)	0
Sinus bradycardia	1 (8)	0
Depression	1 (8)	0

* Treatment related adverse event

Φ Possibly related to 2141-V11
